# A Manual of Dental Anatomy

**Published:** 1882-06

**Authors:** 


*A Manual of Dental Anatomy Human and Comparative.* By Charles S. Tomes, M. A., F. R. S. Second Edition. Publisher: Presley Blakiston, Philadelphia.

It is with pleasure we note the publication of a second edition of this excellent work, which has been entirely revised, and partly rewritten. Many new illustrations have been added until the number in the present edition has reached 191 The chapter on the dental tissues has been altogether revised and its value greatly enhanced. So valuable is this work, that German and French translations have been rendered, thus placing it within reach of all dental practitioners,

Its value as a text book cannot be overestimated and it is highly appreciated wherever it has been introduced. The style in which it appears is very creditable to its American publisher.

